# T2* “Susceptibility Vessel Sign” Demonstrates Clot Location and Length in Acute Ischemic Stroke

**DOI:** 10.1371/journal.pone.0076727

**Published:** 2013-10-11

**Authors:** Olivier Naggara, Jean Raymond, Montserrat Domingo Ayllon, Fawaz Al-Shareef, Emmanuel Touzé, Meriem Chenoufi, Sophie Gerber, Charles Mellerio, Matthieu Zuber, Jean Francois Meder, Jean-Louis Mas, Catherine Oppenheim

**Affiliations:** 1 Department of Neuroradiology, Université Paris-Descartes, INSERM UMR 894, Centre Hospitalier Sainte-Anne, Paris, Ile de France, France; 2 Department of Neurology, Université Paris-Descartes, INSERM UMR 894, Centre Hospitalier Sainte-Anne, Paris, Ile de France, France; 3 Department of Radiology, Saint Joseph Hospital, Paris, France; 4 Department of Neurology, Saint Joseph Hospital, Paris, Ile de France, France; 5 Department of Radiology, The International Consortium of Neuroendovascular Centres, Interventional Neuroradiology Research Unit, University of Montreal, Notre-Dame Hospital, Montreal, QC, Canada; Julius-Maximilians-Universität Würzburg, Germany

## Abstract

**Objectives:**

The aim of our study was to evaluate, in acute ischemic stroke patients, the diagnostic accuracy of the MRI susceptibility vessel sign (SVS) against catheter angiography (DSA) for the detection of the clot and its value in predicting clot location and length.

**Materials and Methods:**

We identified consecutive patients (2006–2012) admitted to our center, where 1.5 T MRI is systematically implemented as first-line diagnostic work-up, with: (1) pre-treatment 6-mm-thick multislice 2D T2* sequence; (2) delay from MRI-to-DSA <3 hrs; (3) no fibrinolysis between MRI and DSA. The location and length of SVS on T2* was independently assessed by three readers, and compared per patient, per artery and per segment, to DSA findings, obtained by two different readers. Clot length measured on T2* and DSA were compared using intra-class correlation coefficient (ICC), Bland & Altman test and Passing & Bablok regression analysis.

**Results:**

On DSA, a clot was present in 85 patients, in 126 of 1190 (10.6%) arteries and 175 of 1870 (9.4%) segments. Sensitivity of the SVS, as sensed by the used protocol at 1.5 T, was 81.1% (69 of 85 patients) and was higher in anterior (55 of 63, 87.3%), than in posterior circulation stroke (14 of 22, 63.6%, p=0.02). Sensitivity/specificity was 69.8/99.6% (per artery) and 76.6/99.7% (per segment). Positive (PPV) and negative predictive value (NPV) and accuracy were all >94%. Inter- and intra-observer ICC was excellent for clot length as measured on T2* (ĸ ≥0.97) and as measured on DSA (ĸ ≥0.94). Correlation between T2* and DSA for clot length was excellent (ICC: 0.88, 95%CI: 0.81–0.92; Bland & Altman: mean bias of 1.6% [95%CI: -4.7 to 7.8%], Passing & Bablok: 0.91).

**Conclusions:**

SVS is a specific marker of clot location in the anterior and posterior circulation. Clot length greater than 6 mm can be reliably measured on T2*.

## Introduction

Recanalization is a powerful predictor of stroke outcome in patients with arterial occlusion treated with either intravenous (IV) recombinant tissue plasminogen activator (rt-PA) or an endovascular approach [1]. Many factors impact the success of recanalization therapy, including clot composition, clot burden [2] and site of clot impaction [3,4]. Recanalization is less frequent in proximal than in distal occlusions [3,4] or when the clot burden is large [2]. In stroke of anterior circulation, response to thrombolysis and clinical outcomes have been best in patients with a small distal middle cerebral artery (MCA) occlusive clot and worst in patients with a large clot occluding the internal carotid artery (ICA) [4-7]. Patients with large clots in proximal vessels may benefit from endovascular interventions, although this hypothesis remains to be proven [8]. Clots can be directly detected with the hyperdense MCA sign on computed tomography (CT) scan. For the purpose of assessment of the amount of clot burden, a CT-angiography-based scale, denominated clot burden score [2], has been proposed, and recently adapted to the T2*-MR sequence [9]. The T2*-MR sequence is sensitive to the susceptibility variation of paramagnetic deoxygenated haemoglobin, which is present in high concentration in acute clots, producing a non-uniform magnetic field, a rapid dephasing of spins, with a dramatic signal loss [10,11]. The presence / length of the susceptibility vessel sign (SVS) on T2*-sequence has never been compared with digitized subtracted catheter angiographic (DSA). The purpose of this study was therefore to compare the presence, location and lengths of clots identified by DSA to SVS in acute ischemic stroke patients.

## Materials and Methods

The study conformed to generally accepted scientific principles and the research ethics standards of our institution and was approved by the Ethics Committee (CPP Ile de France III). The manuscript was prepared in accordance with STARD guidelines. Our institutional review board waived the need for written informed consent from the participants. 

### Cases Identification

The population was nested within a longitudinal cohort of consecutive patients referred to our institution for suspected acute stroke, between January 2006 and July 2012 (n=5811). In our center, MRI has been systematically implemented as first-line diagnostic work-up since 2006. This prospectively maintained database was retrospectively queried to identify all consecutive patients matching the following inclusion criteria: (1) acute ischemic stroke patients; (2) brain MRI performed before treatment decision; (3) DSA performed within 3 hours after MRI completion; (4) contraindication to intravenous fibrinolysis. We recorded the National Institute of Health Stroke Score (NIHSS) at admission, demographic data and the delay from onset to MRI, from MRI to DSA (defined as the delay from the end of MRI to selective microcatheterization beyond the thrombus) and from onset to DSA. Subtypes of ischemic stroke were defined according to Trial of Org 10172 in Acute Stroke Treatment [12], which distinguishes five subtypes of ischemic stroke: (1) large-artery atherosclerosis, (2) cardioembolism, (3) small-vessel occlusion, (4) other determined etiology, and (5) undetermined etiology. 

### Imaging acquisition

#### Brain MRI

All brain MR examinations were performed on a 1.5-Tesla Signa MR Unit (General Electric Healthcare, Milwaukee, WI, USA) using an 8-channel phased-array coil and a previously described standardized stroke protocol [13-16]. The protocol included 6-mm-thick contiguous axial gradient recalled echo T2* (TR=480 ms, TE=13 ms; matrix: 256×224; flip angle: 25°; acquisition time: 1 min 21 sec).

#### Catheter angiogram

All procedures were performed under local anesthesia with IV sedation (n=54) or under general anesthesia (n=31), using 6F catheters introduced by the femoral route. Using a coaxial system, a Renegade (Boston Scientific, Fremont, California, USA) or a Rapid Transit microcatheter (RT; Cordis Endovascular Systems, Miami, Florida, USA) was positioned over a Transend EX 0.014 guidewire (Boston Scientific) through the clot. 

### Image analyses

#### Image quality

Three radiologists (6 months, 1 year and 9 years of experience in stroke imaging, respectively) independently reviewed all T2* images on a dedicated workstation for the presence or absence of SVS. To assess intraobserver reproducibility, one reader re-examined all image sets 2 months later. The overall diagnostic quality of the T2* sequence was scored according to the following three-point grading system: score of 3, excellent quality (arteries clearly depicted and image quality not impaired by artifacts); score of 2, adequate for diagnosis (sharp depiction of arteries; minor artifacts present but not interfering with image interpretation); score of 1: nondiagnostic (depiction of arteries impaired by artifacts). Readers had access to diffusion-weighted images (DWI) but were blinded to all clinical, MR angiography and DSA data. SVS was defined as a dark filling defect within the artery, with a blooming artifact; i.e., the vessel signal was wider than that of other arteries.

#### Clot measurement

On T2*, when an SVS was present, its length was measured and its location noted according to predefined arterial segments. The in-plane length of the clot (M1, P1 segments) corresponded to the distance between the proximal and distal part of the SVS; the length of clot perpendicular to the axial acquisition plane (supraclinoid ICA, basilar artery [BA], A1, A2, M2) was obtained by multiplying the number of cross-sectional locations where the clot was visible by the slice thickness. Inter-observer agreement was studied, followed by a consensus reading session, used to settle disagreements for future comparison with the independent DSA findings. 

On DSA, two other neuroradiologists (5 and 20 years of experience in DSA, respectively), not involved in the care of patients, reviewed DSA series in consensus to assess clot location and extent. The clot length was measured during simultaneous contrast media injection from the guiding catheter and from the microcatheter, positioned distally beyond the clot. This double arterial opacification was performed either by simultaneous manual injection by two physicians, senior and junior interventional neuroradiologists, or by a single manual injection through the microcatheter simultaneously with injection through the guiding catheter using a power injector. Besides the double injection technique, the length of the thrombus was visualized directly, if there was partial flow around the clot or in the case of collateral backflow reaching the site of occlusion by retrograde flow, after a single injection from the guiding catheter. The clot length was defined as the distance from the definite proximal to distal shoulder of the clot in the projection that best elongated the occluded arterial segment. Lesion length was calculated after standardization to the known diameter of a guiding sheath, i.e., the diameter of the microcatheter. Clot length on DSA and on T2* MR sequence were dichotomized in groups >8 mm vs. ≤8 mm, according to and for comparison with earlier CT angiography findings [17]. 

#### Clot location and occlusion grade

**Figure 1:**
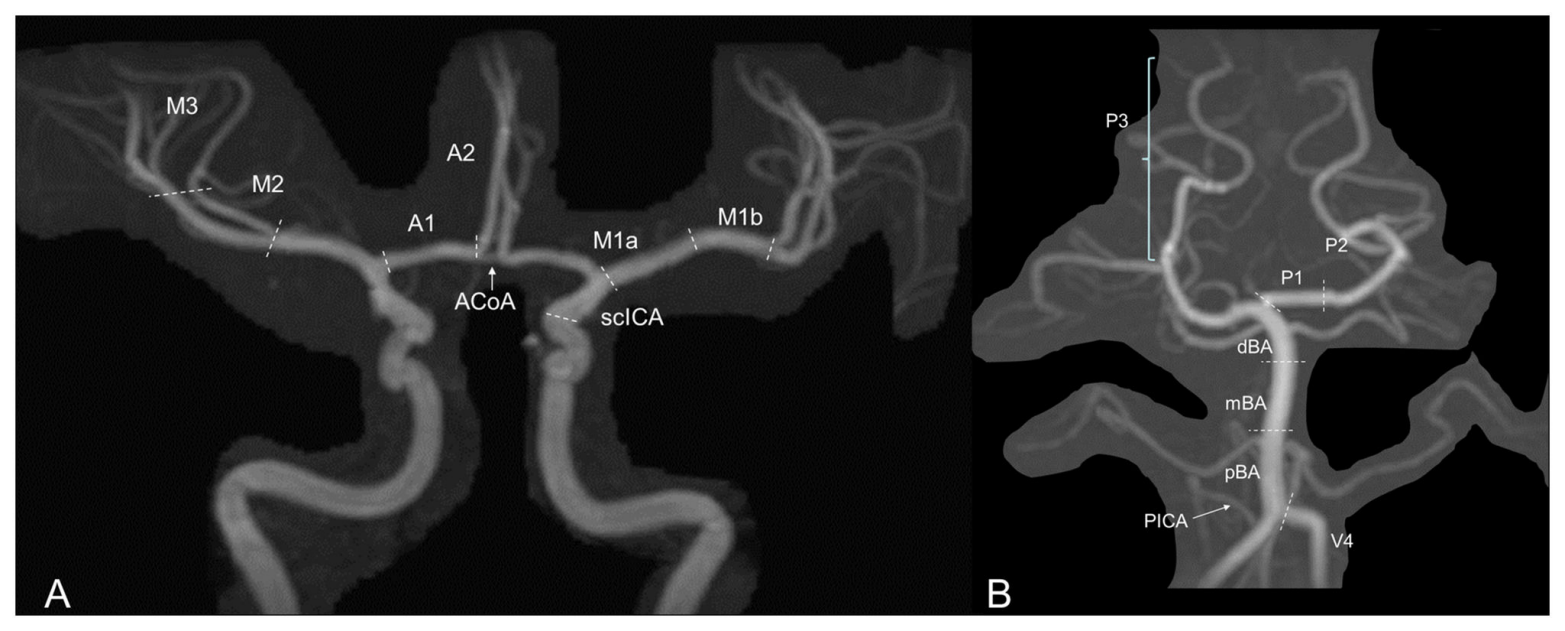
Segmentation of intracranial arteries. Time-of-flight MR angiography of intracranial internal carotid arteries (A) and of the vertebrobasilar arteries (B). Paired arteries were: supraclinoid internal carotid artery (scICA), anterior cerebral arteries (ACA), posterior cerebral arteries (PCA), middle cerebral arteries (MCA), intracranial vertebral arteries (V4), posterior communicating arteries (PComA, not shown), postero-inferior (PICA) cerebellar arteries. Odd arteries were basilar artery (BA) and anterior communicating arteries (AComA). Each ACA was divided into 2 segments: precommunicating anterior cerebral artery (A1) and pericallosal artery (A2). Each PCA was divided into 3 segments: precommunicating PCA (P1) and distal PCA (P2, P3). MCA was divided into 4 segments: proximal part (M1) divided into two segments (M1a, proximal, M1b, distal), insular segment (M2), and opercular segment (M3). Basilar artery was divided into 3 segments (proximal, pBA; medial: mBA; distal: dBA).

We predefined 14 intracranial arteries and 22 arterial segments (Figure 1). T-shaped carotid bifurcation occlusions were diagnosed when the clot involved the supraclinoid ICA (scICA) together with the A1 and M1 segments; L-shaped carotid occlusions were defined by simultaneous involvement of the scICA and either the A1 or the M1 segment. In addition, the occlusion was graded on DSA according to the Thrombolysis in Cerebral Infarction (TICI) classification (Grade 0, no perfusion; Grade 1, penetration with minimal perfusion; Grade 2a, partial filling of the entire vascular territory; Grade 2b, complete filling, but the filling is slower than normal; Grade 3, complete perfusion) [18]. We defined non-occlusive clots as cases in which the contrast material passed beyond the obstruction but failed to opacify the entire cerebral bed distal to the obstruction or opacified the arterial bed distal to the obstruction but with rates of entry and washout that were slower than normal.

### Statistical analysis

Inter-rater agreement in identifying SVS was studied using Kappa (ĸ) statistics and their 95% confidence intervals (_95%_CI). The presence of SVS was compared to DSA per patient, per artery and per arterial segment. Sensitivity, specificity, positive predictive value, negative predictive value, overall accuracy, and positive (PLR) and negative (NLR) likelihood ratios were computed. We also compared SVS and DSA in anterior and posterior circulation strokes in per-patient analysis. 

Inter-rater agreement for clot length was assessed using intra-class correlation coefficient (ICC) and its 95% confidence intervals (_95%_CI). Bland-Altman plots [19] and a Passing & Bablok regression analysis [20] were used to search for systematic differences between measurements. Continuous variables were described as mean ± standard deviation (SD) and non-normally distributed variables were described as median and interquartile range (IQR). Categorical variables and quantitative variables were compared between patients with and without SVS using Mann–Whitney tests or Fisher’s exact tests, as appropriate (MedCalc statistical software, version 9.4.2.0, Mariakerke, Belgium). A two-sided p value of less than 0.05 was considered significant. 

## Results

### Patients

**Figure 2:**
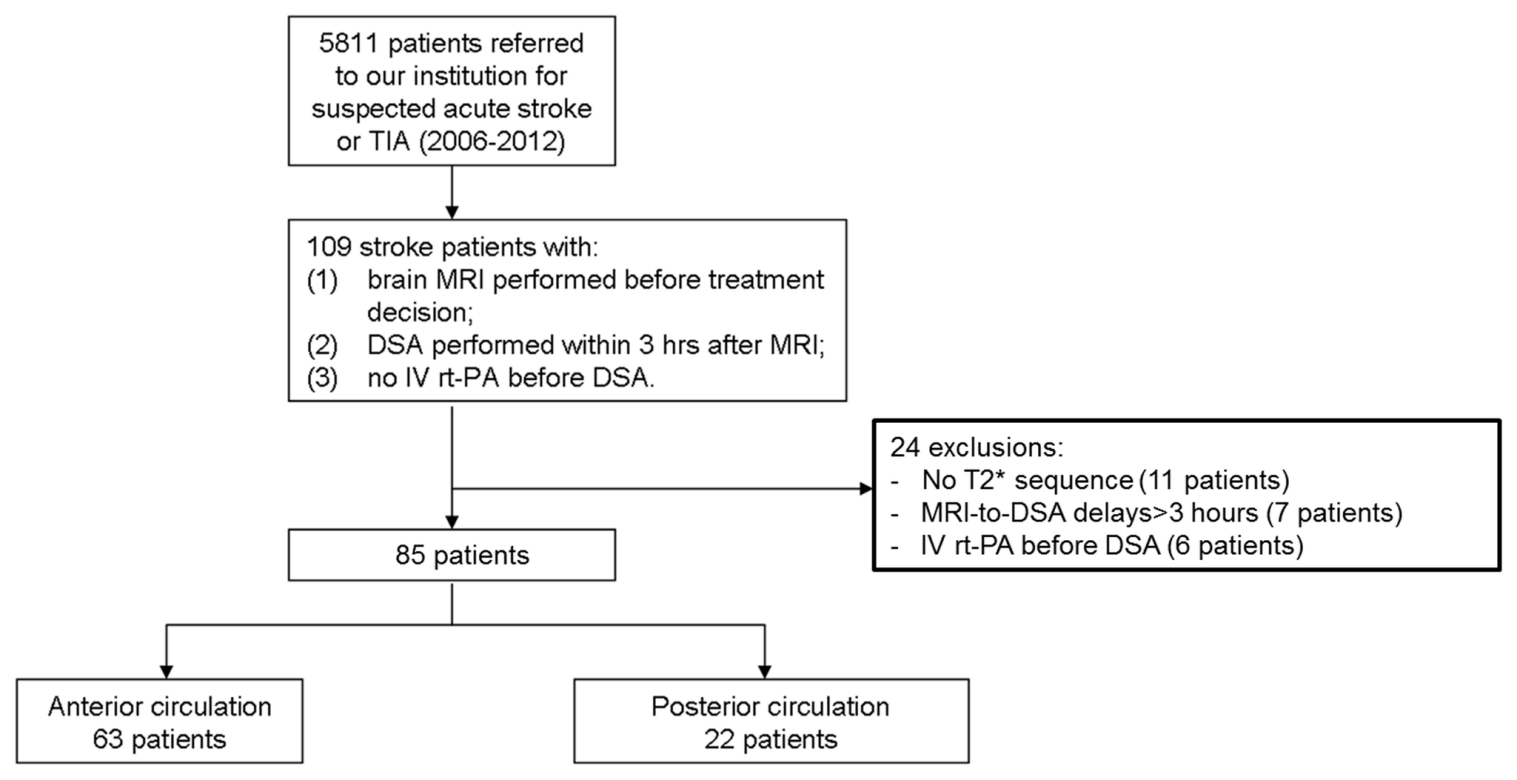
Flowchart of patients.

We identified 403 stroke patients treated for hyperacute stroke (Figure 2), including 85 patients with mechanical thrombectomy alone (48 men; mean age±SD: 56.8±16.2, range: 15–84 years; mean NIHSS±SD: 17.4±7.7, range: 5–37). Contraindication to IV rt-PA included recent surgery (n=8), recent prior stroke (n=4), onset-to-treatment delay >3 h (n=26) before 2009 or >4.5 h (n=24) in 2009 and after, INR greater than 1.7 (n=20), NIHSS <4 (n=3). TOAST classification of ischemic stroke subtypes demonstrated large-artery atherosclerosis in 20 (23.5%) cases, cardioembolism in 31 (36.5%) cases, other determined etiology in 5 (6.0%) cases, and undetermined cause in 29 (34.1%) cases. Onset-to-MRI delay was 216±190 minutes (IQR: 115–255) and MRI-to-DSA delay was 114±55 minutes (IQR: 70-148).

### Clot detection and location on DSA

DSA demonstrated a clot in all 85 patients, in 126 arteries and 175 arterial segments. Sixty-three (74.1%) clots were located in the anterior and 22 (25.9%) in the posterior circulation. In anterior occlusions (63 patients: 96 arteries, 127 segments), 47 clots involved the MCA only, 13 clots caused T-shaped carotid occlusion and 3 clots caused L-shaped carotid occlusion. In the posterior circulation (22 patients: 30 arteries, 48 segments), 13 clots involved the BA only, 8 clots involved simultaneously the BA and posterior cerebral arteries (PCAs), and 1 clot involved the BA, V4 and postero-inferior cerebellar (PICA) arteries.

### Clot detection and location on T2*

**Figure 3:**
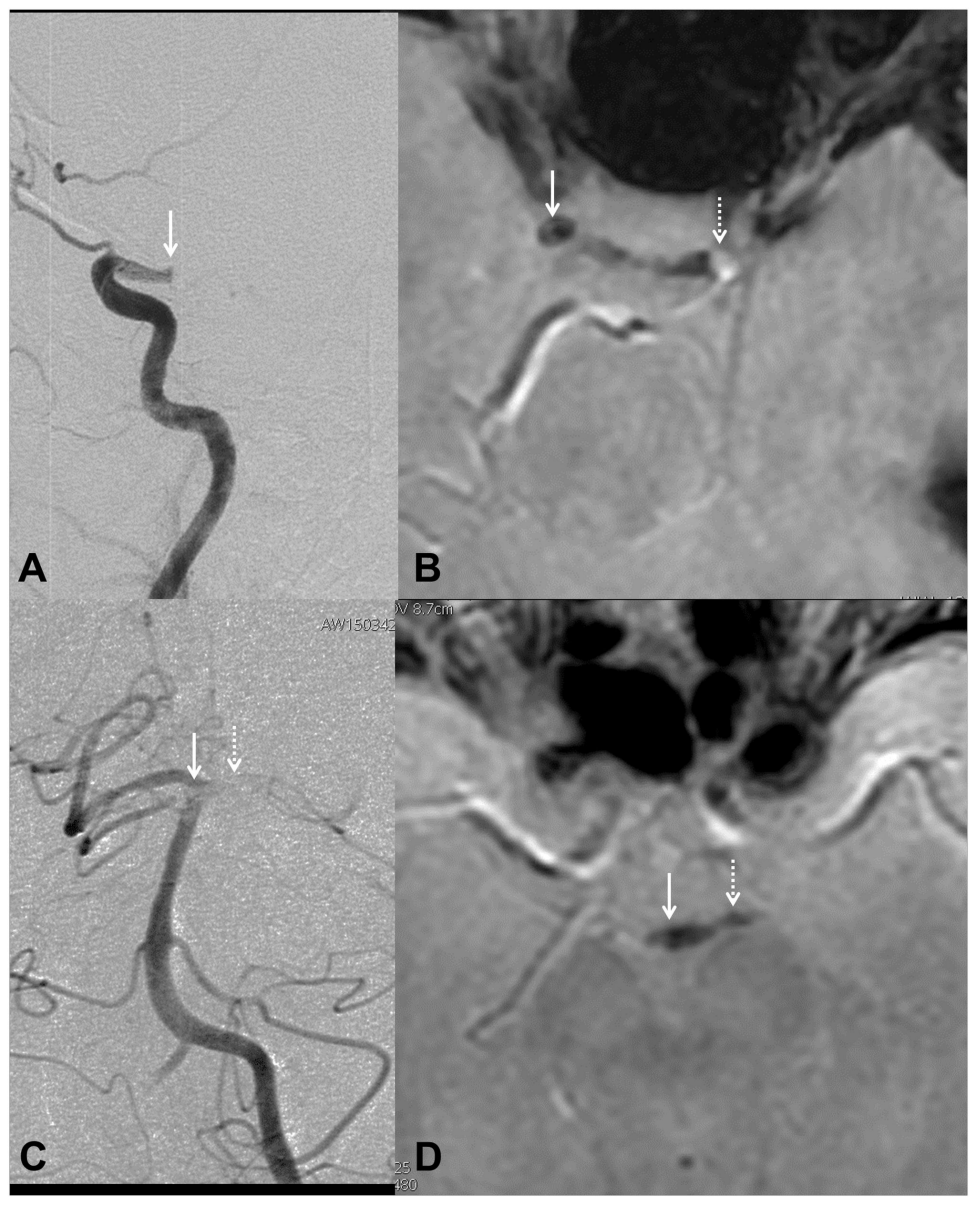
Illustrative examples of complex internal carotid artery terminus and basilar artery occlusion. Right internal carotid artery terminus (ICA) occlusion seen on catheter angiogram (profile projection, A). A susceptibility vessel sign (arrow) was present on T2* sequence in the right supraclinoid ICA, seen as a dramatic signal loss by comparison with contralateral ICA (dotted arrow). Clot of the basilar tip (arrow) and the left P1 segment (dotted arrow) seen on catheter angiogram (frontal projection, C). A susceptibility vessel sign (D) was seen in the same locations (basilar tip, arrow and P1 segment, dotted arrow).

Median quality score of T2* sequence was 2 (IQR: 2–3). Inter- and intra-observer agreement was high for SVS detection (ĸ= 0.83 [0.78 to 0.98] and 0.90 [0.75 to 0.99], respectively). Using DSA as a reference, sensitivity of SVS was 81.1% (69 of 85 patients), 69.8% (88 of 126 arteries) and 76.6% (134 of 175 segments) (Table 1). Patients with and patients without SVS did not differ for age, gender, hypertension, diabetes mellitus, hyperlipidemia, current smoking status, initial NIHSS score or stroke subtypes (data not shown), time from onset to MRI, time from MRI to DSA, or clot location (proximal vs. distal) (Table 2). In the case of SVS on T2*, arteries were significantly more frequently occluded than in the case of a normal T2* sequence (Table 2). There was a higher diagnostic performance of SVS for clot detection in anterior (55 of 63 patients, 87.3%), than in posterior circulation (14 of 22 patients, 63.6%, p=0.02), whereas sensitivity of SVS was not influenced by the proximal or distal location of the clot (p=0.14). False negative and false positive ratings of SVS are shown in Table S1 (supporting information). Among 16 arterial segments with a clot on DSA but no SVS on T2*, five (31.2%) had no occlusion on DSA (TICI=1, grade 2A or 2B) whereas, among 134 arterial segments with SVS, all except three were occluded (p=0.005). There were five distal false positive segments with SVS in A2, M2 and P2 segments, according to DSA findings (Table S1). In all these five cases, a true positive SVS was present in another segment or another artery. In MCA stroke, there was an excellent agreement (ĸ= 0.89 [0.79 to 0.99]) between SVS and DSA for the number of segments involved. All clots involving the proximal and distal M1 segments on DSA were identified using T2*. T- or L-shaped lesions were correctly diagnosed in 10 of 16 cases (Figure 3).

**Table 1:** Diagnostic performance of susceptibility vessel sign (SVS) on T2* according to DSA findings in 85 patients.

	Patient	Artery	Segment
Evaluated on DSA, n	85	1190	1870
Clot on DSA, n	85	126	175
**SVS performance**			
Sensitivity %(_95%_CI)	81.1 (73-89)	69.8 (62-78)	76.6 (70-83)
(n/total n)	69/85	88/126	134/175
Specificity %(_95%_CI)	NA	99.6 (99.2-99.9)	99.7 (99.4-100)
(n/total n)		1185/1190	1690/1695
Positive predictive value	NA	94.6	96.4
Negative predictive value	NA	96.9	97.6
Positive likelihood ratio (_95%_CI)	NA	166.3 (68.8-401.6)	259.6 (107.8-625.2)
Negative likelihood ratio (_95%_CI)	NA	0.30 (0.23-0.40)	0.23 (0.18-0.31)
Pre-test odds	NA	0.11	0.10
Post-test odds	NA	17.6	26.8
Post-test probability	NA	94.6	96.4
Accuracy	NA	96.7	97.5

Abbreviations: n = number, DSA = catheter angiogram, NA = not applicable, _95%_CI = 95% confident intervals

**Table 2:** Comparison between patients with and without susceptibility vessel sign (SVS).

	SVS (n=69)	No SVS (n=16)	*P* values
**Delay**			
Time from onset to MRI, minutes	210.5±191.7	20.5±184.1	0.58
Time from MRI to DSA, minutes	115.4±55.2	104.7±55.2	0.50
**DSA findings**			
Clot length, mm	15.3±7.0	7.6±5.7	0.0002
Complete occlusion (TICI=0)	66	11	0.005
Proximal clot*	59	11	0.14
Anterior circulation	55	8	
Posterior circulation	14	8	0.02
T2* quality score, median (IQR)	2 (2-3)	2 (2-3)	1

Numbers are mean ± standard deviation unless otherwise stated

Abbreviations: DSA = catheter angiogram, TICI = Thrombolysis in Cerebral Infarction classification, IQR = interquartile range.

* A1, M1, P1 segments, basilar artery and supraclinoid internal carotid artery

### Clot length

Inter- and intra-observer intra-class correlation coefficient were high for clot length as measured on T2* (ICC = 0.97 [0.96 to 0.98] and 0.98 [0.96 to 0.99], respectively) and as measured on DSA (ICC = 0.94 [0.91 to 0.96] and 0.94 [0.90 to 0.96], respectively). The mean length of the clot on T2* and on DSA was 17.1±7.2 (range: 6-41) and 15.4±7.1 mm (range: 5-41), respectively. T2* sequence overestimated clot length in 49 of 69 (71%) patients but intra-class correlation coefficient between T2* and DSA for clot length was excellent (ICC, _95%_CI: 0.88, 0.81–0.92). By Passing & Bablok regression analysis (Figure S1, supporting information), the slope was 1.07 [_95%_CI: 1.00–1.20]; the intercept was 0.93 (_95%_CI: -1.20 to 2.00), the correlation coefficient (R) was 0.91; the cusum test did not demonstrate significant deviation from linearity (p>0.10). The Bland-Altman plot showed a mean bias of 1.6% (_95%_CI:-4.7 to 7.8%). 

**Figure 4:**
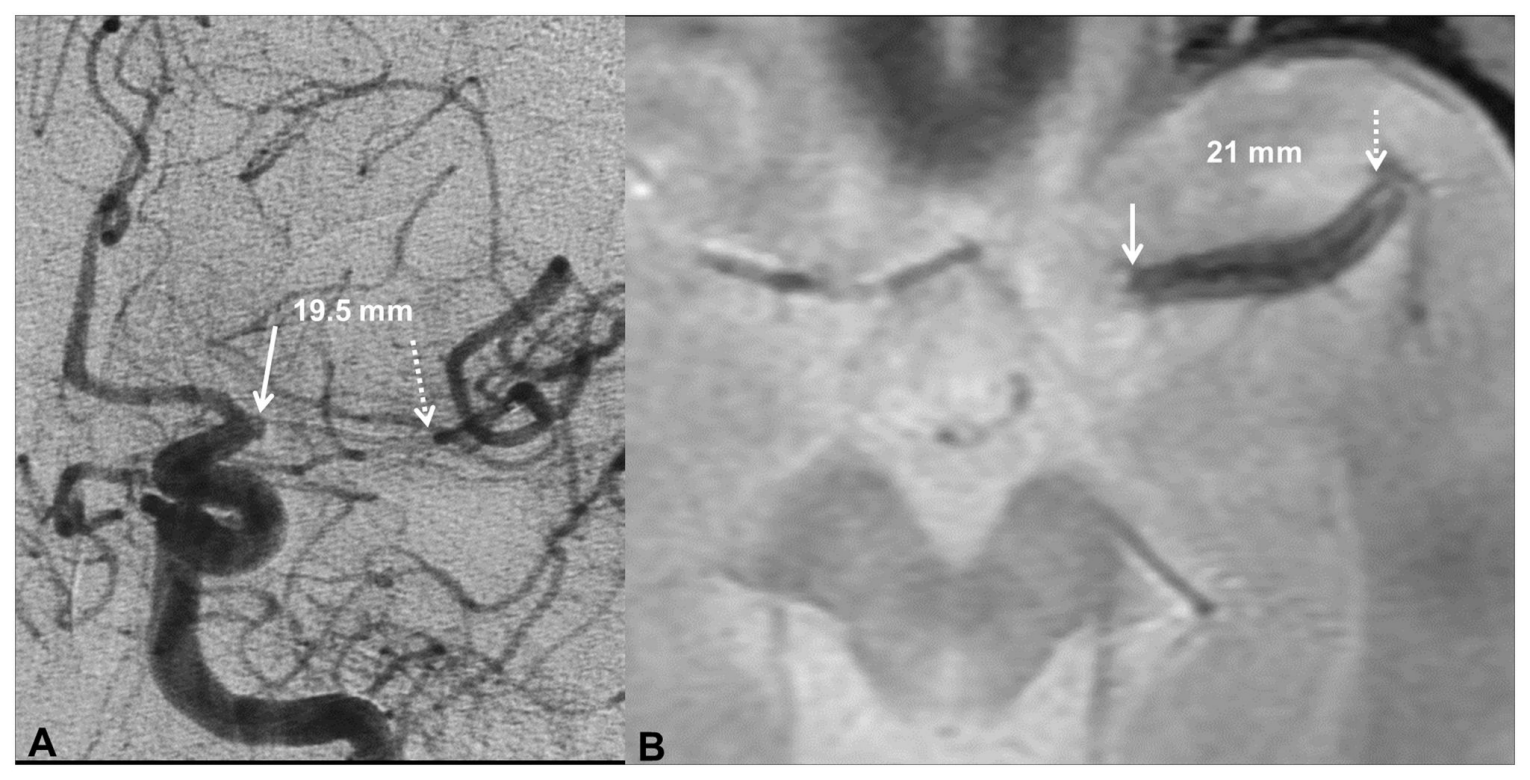
Illustrative example of clot length measurement on DSA and T2*. Occlusive MCA clot on frontal projection of catheter angiogram (A) and T2* (B).

In the case of SVS on T2*, clot length on DSA was significantly greater and arteries were significantly more frequently occluded than in the case of a normal T2* sequence (Table 1). Sensitivity of SVS for clot length >8 mm was 94.9% (_95%_CI: 89.3 to 100%). Only 6 of 62 clots longer than 8 mm on DSA were misclassified using measurement on T2* (Figure 4).

## Discussion

We found that clot location and length could be reliably assessed using the SVS, with a high concordance with DSA findings. We also confirmed that the SVS is a relatively sensitive and specific marker of acute clot, not only in MCA occlusions, as previously reported [21], but also in ICA occlusions, or in occlusion in the posterior circulation. SVS diagnostic performance was not influenced by the proximal or distal location of the clot. 

The treatment of arterial occlusion aims at restoring blood flow by intravenous fibrinolysis administered within 4.5 h after symptom onset [22]. Chemical clot dissolution depends on its length and surface area exposed to blood flow and to the lytic agent at its proximal and distal ends [4]. Riedel et al. demonstrated that clots with a length >8 mm, as measured on CT angiography, did not recanalize with IV rt-PA alone [17]. We recently demonstrated that a semi-quantitative clot burden score on T2* sequence was predictive of recanalization within 24 h and clinical outcome at 3 months in a cohort of acute anterior circulation stroke patients treated with IV thrombolysis [9]. Interestingly, we showed here that clot length can be directly measured on axial T2* sequence. Furthermore, the sensitivity of SVS for clots longer than 8 mm on DSA was almost perfect. Thus, SVS might help to identify patients unlikely to recanalize with IV-tPA alone and that might be candidates for mechanical thrombectomy or add-on antithrombotic drugs at the acute stage of stroke. Radiologists have been reluctant to measure signal drop on T2* because of the commonly held view that the clot-related magnetic susceptibility effects overestimate the true size of the clot. The present study challenges this conception, since we found an excellent agreement between the measurement of detected clots longer than 6 mm using T2* and DSA. 

Our results suggest that clot locations as defined with the SVS closely matched those obtained with the standard of reference, i.e., DSA with simultaneous contrast injection within and beyond the clot. In MCA occlusion, SVS distinguished between partial and complete involvement of the M1 segment. Similarly, T- and L-shaped carotid occlusions were in most cases diagnosed using SVS. Among all anterior arterial occlusions, such complex ICA occlusions carry the worst prognosis and are unlikely to recanalize with IV rt-PA alone [4,23]. Thus, an early and reliable demonstration of T- or L-shaped extension of the clot on routinely used axial T2* sequence may help guide therapeutic decisions [24-26]. Hence, in future interventional stroke trials, clot length and location, as defined on T2* sequence, could help select homogenous groups of patients with a high clot burden or an unfavorable clot impaction site, who might benefit from more aggressive recanalization strategies.

The sensitivity and specificity of the SVS were however imperfect. False negative studies were mainly encountered in cases of partial occlusion, thereby suggesting that not only the clot composition but also the occlusion status might contribute to the visibility of the SVS. The lack of SVS sensitivity in the presence of partial flow was previously described in the case of partial recanalization after intravenous fibrinolysis [27]. Experimental data previously suggested that the entrapment of red blood cells and the concentration of platelets and fibrin may be crucial in the extent of SVS caused by a clot [25,28]. A large SVS was reported in the presence of red blood cell-rich clots whereas SVS was lacking in fibrin-dominant clots [29]. Residual flow or complete stasis has previously been invoked in determining clot composition [30,31]. Hence, some authors considered that thrombus composition is not just the cause, but also the consequence of impaired flow, since the red blood cell content was augmented by stasis of the flow in the case of complete occlusion [29].

Clots might also have been missed because of susceptibility artifacts at the skull base. In addition, partial volume effect on 6-mm-thick 2D multislice T2* sections might account for the imperfect sensitivity for short clots, since we demonstrated a higher sensitivity of SVS for longer clots. On the other hand, false positive SVS might be explained by longer MRI-to-DSA delays, given that spontaneous recanalization might occur as time elapses. Possible improvements may include the use of thinner slice images, the use of higher field strength and new MRI sequences. Another interesting approach to evaluate clot length and location is time-of-flight MR angiography (TOF MRA). TOF MRA detects embolic occlusion of arteries in patients with acute ischemic stroke due to the absence of blood flow in the occluded vessel. However, TOF MRA does not fully capture clot length, especially its distal part. Recently, Radbruch et al. demonstrated that 3D susceptibility weighted imaging (3D SWI) directly enables clot visualization based on the hypointense SVS in the occluded vessel [32]. The use of 3D SWI yielded increased sensitivity to susceptibility inclusions such as deoxyhemoglobin within the arterial clot compared to conventional T2*-weighted imaging [33,34]. Another limitation of clot imaging obtained using T2* or 3D SWI is that SVS is dependent on imaging parameters, including echo time, field strength, and voxel size. Recently, a novel quantitative susceptibility mapping (QSM) technique for postprocessing gradient echo data was applied successfully as a means of obtaining a universal measurement of the burden of cerebral microbleeds or brain hematoma, thereby eliminating the T2* dependence on imaging parameters and providing measurement independent of imaging parameters [33,35].

However, acquisition time is a major issue for optimal treatment workflow, since endovascular or intravenous treatment has to be applied as soon as possible in patients with acute stroke. Routine clinical implementation of 3D SWI may be difficult due to the longer acquisition time (between 5 and 10 minutes). Further studies are needed to investigate whether acquisition time in stroke imaging can be reduced, for example by the simultaneous acquisition of TOF and SWI [36], or by the use of 2D SWI processed from standard T2* imaging as used in our study [37]. 

We presumed that the SVS visualizes the clot material and we cannot rule out the possibility that stagnating blood in front of or behind the clot and/or arteriosclerotic vessel walls may contribute to the signal loss. This question arose especially in the vertebrobasilar system, where arteriosclerosis is more frequent and considering the fact that a T2* sequence with a slice thickness of 6 mm was used. Because there is no roadmap of the site of the occlusion and the vessels behind, the microcatheter is navigated blind through the site of occlusion. In the case of M1 occlusion it may be difficult to define the end of the clot. Consequently, to estimate the end of the clot, we performed multiple injections while withdrawing the microcatheter step by step close to the end of the clot. One may argue that the thrombus might have been dislodged during these multiple passages of the microcatheter.

This study has several limitations. First, because the population consisted mainly of patients referred for intra-arterial therapy, biased for more severe strokes from more proximal occlusion, mainly long clots were measured and compared to DSA. Thus, our results are hardly generalizable to smaller clots and finding a good agreement on long-clot length between T2* sequence and DSA is no guarantee of accurate length measurement of smaller clots. Second, because readers were aware that all stroke patients had been referred for DSA, sensitivity might have been artificially increased. Third, MRI acquisition parameters and type of T2* sequence may greatly influence the sensitivity of detection of the SVS [33]. We, like others [27,38], used a conventional gradient echo acquisition, a choice that may have increased sensitivity by comparison with studies using echo-planar acquisition [21,39]. In addition, the sensitivity of SVS may depend on the onset-to-MRI delay, which was shorter in our study than in others. However, we demonstrated that sensitivity of the SVS was not influenced by onset-to-MRI delay. Finally, although our results apply only to centers that use MRI as first-line imaging workup in acute stroke, and as such have limited applicability, large centers are increasingly implementing acute stroke MRI given its acknowledged safety and clinical utility.

In conclusion, our study suggests that SVS predicted precise clot location, particularly complex terminus and L-type carotid occlusion. Length of the clot on DSA and on T2* were comparable, suggesting that clot length can be directly measured on T2*. Consequently, the SVS could help to better identify patients with long clots and make an informed decision. 

## Supporting Information

Figure S1 Clot length: correlation between DSA and T2*. By Passing & Bablok regression analysis (A), the slope, the intercept and the correlation coefficient were, respectively, 1.07 (_95%_CI: 1.00–1.20), 0.93 (-1.20–2.00) and 0.91; the cusum test did not demonstrate significant deviation from linearity (p>0.10). Dashed line, deming regression; dotted line, 1:1 line. The Bland-Altman plot (B) showed a mean bias (solid line) of 1.6% (_95%_CI: -5.4–8.6%, dashed line).
(TIF)

Table S1 **Location of clot in false negative and false positive cases of susceptibility vessel sign (SVS).**
(DOC)
