# Supplementary material for: T2* “Susceptibility Vessel Sign” Demonstrates Clot Location and Length in Acute Ischemic Stroke
Source: PLoS One. 2013 Oct 11;8(10):e76727. doi: 10.1371/journal.pone.0076727 (PMC3795632; doi:10.1371/journal.pone.0076727)
Supplement: Table S1 — Location of clot in false negative and false positive cases of susceptibility vessel sign (SVS). (DOC) [file pone.0076727.s002.doc]

**Table S1. Location of clot in false negative and false positive cases of susceptibility vessel sign (SVS)**

| Location | False negative  n=38 segments | False positive  n=5 segments |
| --- | --- | --- |
| supraclinoïd artery | 6 | 0 |
| anterior cerebral artery | 9 | 2 |
| A1 segment | 9 | 0 |
| A2 segment | 0 | 2 |
| middle cerebral artery | 7 | 2 |
| M1 segment | 3 | 0 |
| M2 segment | 3 | 2 |
| M3 segment | 1 | 0 |
| Basilar artery | 11 | 0 |
| proximal | 1 | _ |
| medial | 4 | _ |
| distal | 6 | _ |
| Posterior cerebral artery | 5 | 1 |
| P1 segment | 2 | 0 |
| P2 segment | 2 | 1 |
| P3 segment | 1 | 0 |
